# Case of Cirrhosis of the Liver and Bronzed Skin

**Published:** 1857-07

**Authors:** Charles Fricke

**Affiliations:** Baltimore


ART. V.—*Case of Cirrhosis of the Liver, and Bronzed Skin.* By CHARLES FRICKE, M.D., of Baltimore.

MICHAEL BATEMAN, white, born in Ireland, æt. 25, five feet five inches high, moderately fleshy, and whose previous occupation was a laborer in one of the lower counties of the State, was admitted to the prison in April, 1856. He admitted that he drank occasionally, but denied being intemperate, and his health, excepting occasional attacks of malarious fever, had been good. Nothing particular was remarked about his appearance, but his hands. These were fully one-third larger than usual, evidently from hypertrophy of the skin, which, along the fingers, was disposed somewhat in folds, and which appearance I concluded to be due to elephantiasis. He was put to medium work, and attracted no attention from me, until the first of September, following. I found then that he had failed in strength; that he complained of nausea, and distension about the epigastric region, the latter due to gas; more or less headache and constipation, but no fever. A few days' rest served in a measure to relieve this condition, and he returned to work. At the end of the month he again entered the hospital with the same symptoms, but accompanied with jaundice and a sense of weight rather than pain over the liver. He was ordered taraxicum and blue mass, and in a few days was again relieved. This recurred from time to time until the middle of December, when he became a permanent inmate of the hospital. The jaundice was now universal, pulse 80, and of ordinary volume; no perceptible enlargement of liver; lungs and heart sound; digestive function good; emaciation moderate; and urine high-colored. His principal complaint was of lassitude and an uneasy sensation in the head. As the case progressed, the principal phenomena exhibited were dropsy, hemorrhage, black urine, and bronzed skin. The first of these, dropsy, appeared in the form of ascites, about the middle of February, and had attained a very considerable size at the time of his death, but the abdominal veins were never much enlarged; and during the last few weeks, his legs became œdematous. The first hemorrhage occurred on the 14th of April, from the intestinal canal, as well as from the lungs and stomach. The whole amounted to nearly a quart. These recurred three or four times during the succeeding two weeks. I first remarked the black urine in March. It continued till his death. The average amount was 30oz., and sp. gr. 1·020. By direct light this fluid resembled bile, but by transmitted light, was of a deep purple hue. It contained no very great excess of bile, and the change was found to be due to an enormous amount of the coloring matter, termed by Heller, *urrhodin*. The fecal discharges for the last six months were hard, rounded, and white balls, somewhat resembling that of dogs.

The bronzed skin, the most interesting phenomenon, and the one to which I wish to direct attention more particularly, was first remarked in January. At the commencement it was not well defined, nor more decided than is often observed in cases of cirrhosed liver, but after a time, the tinge became so deep as to attract decided attention. It was limited to the forehead, face, and neck, and almost as deep in hue, as is formed in the livers of remittent fever patients. One fact is worthy of notice. The demarcation between this color and that of the rest of the body was not clearly defined, but they merged into each other by degrees. He died April, 29, 1857, after a convulsion.

The autopsy was made by Dr. Johnston and myself, seven hours after death. Bronze hue not so well marked, jaundice universal and of a deep lemon tint, emaciation moderate, and legs œdematous. Head and chest not examined. The abdominal cavity contained about one gallon of serous fluid, and the intestinal canal was filled with dark liquid blood. Kidneys congested and somewhat enlarged, otherwise healthy. Suprarenal capsules of normal size, hue, and consistence; no alteration whatever observable. The liver, one-third smaller than usual, was of a bronze hue externally. Everywhere its surface was irregular, and in some places nodulated, particularly the left lobe. Internally the fibrous texture was abnormally developed, and the outline of the lobules clearly defined. The cut surface, but not the exterior of the organ, was uniformly of a light apple or pea-green color. Under the microscope, nothing was perceptible but the hypertrophied fibrous tissue and the cells filled with the same coloring matter. The gall-bladder contained about ℥ss of light green bile. Although various shades are spoken of by different pathologists, I can find in no work this peculiar color of the liver alluded to.

